# Localized eruption of glabella and eyebrows

**DOI:** 10.1016/j.jdcr.2024.08.008

**Published:** 2024-08-31

**Authors:** Celestina Okoye, Kai Brady, Jennifer Shastry

**Affiliations:** Department of Dermatology, McGaw Medical Center of Northwestern University, Chicago, Illinois

**Keywords:** Arndt-Gottron disease, monoclonal gammopathy, mucinosis, sclerodermoid lichen myxedematous, scleromyxedema

## History

**Figure 1:**
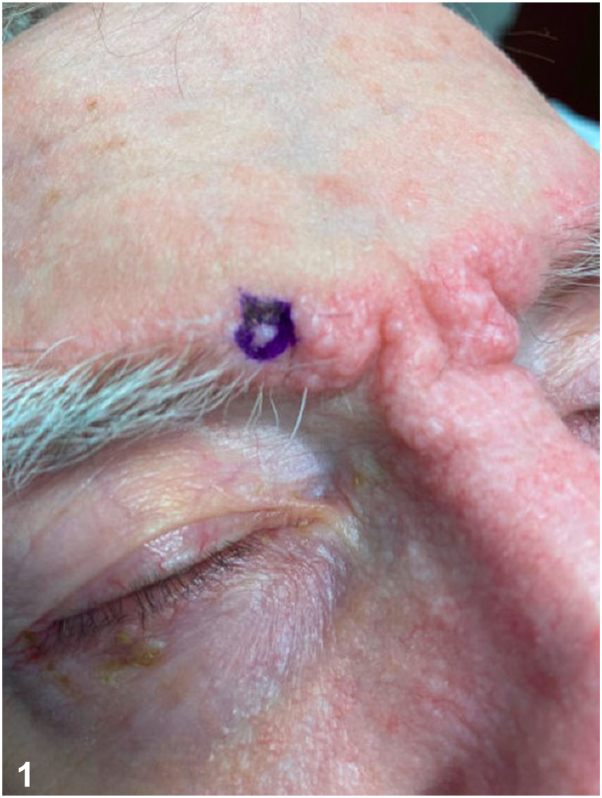

**Figure 2:**
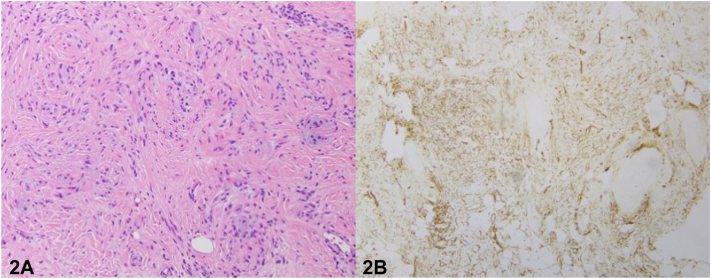

A 79-year-old male with a past medical history significant for atrial fibrillation, cerebrovascular accident, IgG lambda smoldering multiple myeloma, lumbar radiculopathy, osteoporosis, and rheumatoid arthritis presented with a 4-month history of a progressive eruption over the eyebrows and glabella (Fig 1). Physical examination demonstrated pink telangiectatic papules coalescing into plaques of the bilateral eyebrows and glabella. A punch biopsy was performed (Fig 2, *A*), and a CD34 stain is pictured (Fig 2, *B*).

**Question 1: What is the most likely diagnosis based on the history, clinical, and histologic findings?**

A. Sebaceous hyperplasia
B. Phymatous rosacea
C. Amyloidosis
D. Scleromyxedema
E. Cutaneous T-cell lymphoma

**Answers:**

A. Sebaceous hyperplasia – Incorrect. Sebaceous hyperplasia is a benign condition commonly appearing on the forehead and cheeks. Clinically, sebaceous hyperplasia appears as small yellow papules with a central dell. The histologic findings of sebaceous hyperplasia are enlarged but otherwise normal-appearing sebaceous glands surrounding dilated follicle(s).
B. Phymatous rosacea – Incorrect. Rosacea is characterized by erythematous papules and pustules. While thickening of the skin is also seen in scleromyxedema, in phymatous rosacea it is due to hyperplasia or fibrosis of sebaceous glands. The histologic features of phymatous rosacea depend on the distinct subtype and stage of disease.
C. Amyloidosis – Incorrect. Cutaneous amyloidosis has variable clinical presentation that includes macular, papular, and nodular forms. The histology of amyloidosis depends on the subtype; however, all subtypes involve amyloid or amyloid-like protein deposition in the dermis.
D. Scleromyxedema – Correct. Scleromyxedema causes a papular and sclerodermoid eruption on the skin and may cause leonine facies.^1^ Papules are typically firm and waxy in appearance and may be arranged in a linear fashion. The histopathologic features include a triad of mucin deposition, proliferation of CD34+ fibroblasts, and fibrosis.^2^
E. Cutaneous T-cell lymphoma – Incorrect. Cutaneous T cell lymphoma may present with leonine facies; however, the lack of an atypical lymphocytic infiltrate on histopathology excludes this diagnosis.

**Question 2: What is the pathophysiology of this disease?**

A. Fibroblasts are stimulated to increase mucin production and deposition in the skin.
B. High glucose levels result in glycosylation of collagen and excessive mucin deposition between collagen bundles.
C. Gadolinium interacts with macrophages to stimulate profibrotic cytokine release and collagen formation, leading to tissue fibrosis.
D. Autoantibody-driven T cell activation and profibrotic cytokine release result in excess collagen deposition and microvascular dysfunction.
E. Hyperplasia and fibrosis of the sebaceous glands.

**Answers:**

A. Fibroblasts are stimulated to increase mucin production and deposition in the skin – Correct. While the exact mechanism of scleromyxedema is unclear, the proposed mechanism involves the stimulation of the production of hyaluronic acid and prostaglandin E by fibroblasts by unknown hematogenous factors.^3^^,^^4^
B. High glucose levels result in glycosylation of collagen and excessive mucin deposition between collagen bundles – Incorrect. This describes the pathophysiology of scleredema diabeticorum.
C. Gadolinium interacts with macrophages to stimulate profibrotic cytokine release and collagen formation, leading to tissue fibrosis – Incorrect. This is the proposed pathophysiology of nephrogenic systemic fibrosis.
D. Autoantibody-driven T cell activation and profibrotic cytokine release result in excess collagen deposition and microvascular dysfunction – Incorrect. This is the etiology of systemic sclerosis.
E. Hyperplasia and fibrosis of the sebaceous glands – Incorrect. This is the pathophysiology of sebaceous hyperplasia.

**Question 3: Which of the following aspects of this patient’s past medical history is most strongly associated with this condition?**

A. Cerebrovascular accident
B. Rheumatoid arthritis
C. Atrial fibrillation
D. Monoclonal gammopathy
E. Lumbar radiculopathy

**Answers:**

A. Cerebrovascular accident – Incorrect. Cerebrovascular incidents are not a common extracutaneous manifestation of scleromyxedema.
B. Rheumatoid arthritis – Incorrect. Rheumatologic disorders were the second most common (25%) extracutaneous manifestation of scleromyxedema in a multicenter study.^5^ Rheumatologic manifestations commonly include inflammatory myopathy and rheumatoid arthritis.
C. Atrial fibrillation – Incorrect. Cardiac involvement was the third most common (22%) extracutaneous manifestation of scleromyxedema in a multicenter study.^5^ Reported cardiac manifestations include congestive heart failure, heart block, and pericardial effusion.
D. Monoclonal gammopathy – Correct. Monoclonal gammopathy was present in 27 out of 30 patients with scleromyxedema in a multicenter study.^5^ Extracutaneous manifestations of scleromyxedema are common yet variable. Monoclonal gammopathy has the strongest association with scleromyxedema.
E. Lumbar radiculopathy – Incorrect. Lumbar radiculopathy is not a common extracutaneous manifestation of scleromyxedema.

## Conflicts of interest

None disclosed.
